# A deep ensemble learning approach for squamous cell classification in cervical cancer

**DOI:** 10.1038/s41598-025-91786-3

**Published:** 2025-03-01

**Authors:** Jayesh Gangrade, Rajit Kuthiala, Shweta Gangrade, Yadvendra Pratap Singh, Manoj R, Surendra Solanki

**Affiliations:** 1 Department of Artificial Intelligence & Machine Learning, School of Computer Science & Engineering, Manipal University Jaipur, Jaipur, Rajasthan India; 2 Department of Information Technology, School of Computer Science & Engineering, Manipal University Jaipur, Jaipur, Rajasthan India; 3 Department of Computer Science & Engineering, Manipal Institute of Technology Manipal, Manipal Academy of Higher Education, Udupi, Karnataka India

**Keywords:** Cervical Cancer, Image Classification, AlexNet, SqueezeNet, Ensemble Learning, Cancer, Cancer prevention

## Abstract

Cervical cancer, arising from the cells of the cervix, the lower segment of the uterus connected to the vagina-poses a significant health threat. The microscopic examination of cervical cells using Pap smear techniques plays a crucial role in identifying potential cancerous alterations. While developed nations demonstrate commendable efficiency in Pap smear acquisition, the process remains laborious and time-intensive. Conversely, in less developed regions, there is a pressing need for streamlined, computer-aided methodologies for the pre-analysis and treatment of cervical cancer. This study focuses on the classification of squamous cells into five distinct classes, providing a nuanced assessment of cervical cancer severity. Utilizing a dataset comprising over 4096 images from SimpakMed, available on Kaggle, we employed ensemble technique which included the Convolutional Neural Network (CNN), AlexNet, and SqueezeNet for image classification, achieving accuracies of 90.8%, 92%, and 91% respectively. Particularly noteworthy is the proposed ensemble technique, which surpasses individual model performances, achieving an impressive accuracy of 94%. This ensemble approach underscores the efficacy of our method in precise squamous cell classification and, consequently, in gauging the severity of cervical cancer. The results represent a promising advancement in the development of more efficient diagnostic tools for cervical cancer in resource-constrained settings.

## Introduction

Cervical cancer is a global health concern that disproportionately affects women worldwide. By automating the interpretation of Pap smear images, we can minimize pathologists’ workloads and find precancerous and cancerous abnormalities earlier. This can lead to timely intervention, perhaps saving lives.Pap smear screening is a microscopic examination of cervical cells to detect any abnormalities^1^. This method, while effective, is time-consuming and requires specialized staff. It is useful in detecting alterations produced by the human papillomavirus vaccine (HPV), which, if left untreated, can lead to cervical cancer, allowing for the identification of precancerous or cancerous cells, as well as non-malignant diseases such as infection or inflammation^2^. This is the benefit of HPV vaccination in women who have had a hysterectomy for high-grade cervical precancer or early-stage cervical cancer. The researchers reviewed the medical records of 77 patients who acquired lower genital tract dysplasia following surgery. They discovered that the HPV vaccine could have avoided a considerable number of these cases^3^.However,It provides minimal protection to those who are already affected. To overcome the limitations of manual approaches, computer-aided diagnostic (CAD) systems have emerged as promising cervical cancer screening tools^4–6^.

In India, cervical cancer represents a particularly significant concern, with a staggering. One in every five patients worldwide comes from the country. The economic impact of both medical and non-medical expenses, as well as lost productivity, highlights the need for effective preventative and screening measures^7^. Cervical cancer is the most common malignancy among Indian women, accounting for 72,825 deaths each year and 26.7% of the global total. The age-adjusted incidence rate ranges from 8.8 to 10.1 per 100,000. Low-and middle-income countries face challenges in conducting comprehensive screening programs due to a lack of qualified healthcare staff and limited resources^6,8^.

These systems use image processing and machine learning approaches to examine digital images of cervical cells. In recent years, deep learning, a type of machine learning, has demonstrated extraordinary effectiveness in a variety of medical image analysis tasks, including cervical cancer detection^7,9,10^. Deep learning algorithms, such as Convolutional Neural Networks (CNNs), can automatically learn hierarchical features from images, allowing for precise classification of cervical cell abnormalities.Cervical cancer is a major global health concern, especially in developing countries. Early detection is critical for successful treatment and better patient outcomes. Traditional screening approaches, such as Pap smears, are highly dependent on the expertise of cytotechnologists or pathologists, which can be subjective and prone to human error. This research uses deep learning and Pap smear images to classify cervical cancer. We use CNN, SqueezeNet, and AlexNet to classify cervical cancer cells into five distinct categories: “dyskeratotic,” “koilocytotic,” “metaplastic,” “parabasal,” and “superficial-intermediate”. Additionally, an ensemble learning technique combines individual model predictions to improve overall accuracy.

Research objectives can be summarized as follows:

- The study aimed to design a system that could automatically categorize squamous cells from Pap smears into different classes representing the severity of cervical cancer.
- By employing deep learning techniques like CNN, AlexNet, and SqueezeNet, the goal was to achieve a more precise categorization of squamous cells compared to manual analysis..
- The research explored combining the strengths of multiple deep learning models by ensemble technique to achieve a higher overall accuracy in classifying squamous cells.

The paper is structured as follows: Section 2 provides an overview of the literature on cervical cancer. Section 3 outlines the proposed methodology, which employs ensemble learning techniques incorporating CNN, AlexNet, and SqueezeNet. Section 4 presents the dataset, evaluation parameters, and results,Section 5 discuss the limitation of the Ensemble method, Finally, Section 6 offers concluding remarks.

## Literature review

The literature review provides an extensive overview of various techniques and models employed for the classification and detection of cervical cancer. It is evident that a range of approaches utilizing computer-based algorithms, deep learning, and ensemble learning have been explored in this domain. Each study leverages different methodologies, datasets, and models to achieve accurate results.

Mango et al.^11^introduced a solution for detecting cancerous cells in cervix samples, integrating a conventional Pap smear test with an artificial neural network (ANN) model. Sukumar and Gnanamurthy (2016)^12^presented an automated diagnostic method based on magnetic resonance imaging scans. Their hybrid classifier, combining SVM and adaptive neuro-fuzzy interface technology, achieved remarkable accuracy. Bora et al. (2016)^13^employed a deep convolutional neural network for image identification, enhancing accuracy through feature selection tasks. Their comparative study of LSSVM and SoftMax regression classifiers highlighted substantial improvements in classification rates. Hyeon et al. (2017)^14^used CNNs and machine learning classifier-based models for classifying cervical MRIs, showcasing the effectiveness of feature extraction using the VGG16 algorithm. Promworn et al. (2019)^15^conducted a deep learning comparative analysis of cervical cytopathology images, with DenseNet161 emerging as the most accurate model among five deep learning approaches. ColpoNet drew inspiration from DenseNet for its computationally efficient framework^16^. Parikshit Sanyal et al.^17^developed a CNN for detecting abnormal foci in traditional cervical smears, achieving a high diagnosis accuracy. Karunakaran et al. (2020) proposed ultrasensitive surface enhanced Raman scattering (SERS) for predicting cervical cell samples’ pathology with commendable accuracy^17^. Taha et al. (2017) emphasized the benefits of employing pre-trained CNN architectures, such as AlexNet, for classification tasks^18^. Kudva et al. (2020) introduced a hybrid transfer learning system, utilizing AlexNet and VGG-16 features to improve cervix image identification. Their results demonstrated substantial gains in classification accuracy^19^. Xue et al. (2020) utilized Ensemble Transfer Learning (ETL) to classify cervical histopathology images, achieving impressive accuracy scores^20^. Chen et al. (2020) explored the potential of CNNs and transfer learning in histopathological image analysis, yielding a high classification accuracy^21^. Ghoneim et al. (2020) demonstrated the effectiveness of CNN-based approaches for detecting and categorizing cervical cancer cells. Their use of CNN models in tandem with ELM classifiers showcased promising results on the Herlev database^22^. Arifianto et al.^23^ applied CNN deep learning methods to a diverse dataset, achieving notable accuracy in identifying cervical lesions. Hussain et al.^24^ proposed several models based on deep convolutional neural networks, reporting impressive accuracy scores across different datasets. Kang et al.^25^ explored the use of Raman spectroscopy and a novel hierarchical neural network (H-CNN) to accurately identify various stages of cervical cancer in tissue samples. H-CNN outperformed traditional methods in accuracy, stability, and sensitivity, achieving over 94 % accuracy in classifying tissues. This suggests H-CNN could be a promising tool for early and precise cervical cancer diagnosis, potentially improving patient outcomes. Youneszade et al.^26^ highlights the increasing role of deep learning in tackling cervical cancer’s burden, particularly in resource-limited areas. It effectively points out the limitations of traditional screening methods and how DL-based computer-aided diagnostics offer the promise of improved accuracy and early detection. By reviewing relevant techniques, architectures, and segmentation methods, it provides a valuable overview of the current state-of-the-art for DL in cervical cancer screening. Finally, it emphasizes the need for further research and offers avenues for future exploration in this crucial field. Overall, this is a concise and informative summary that captures the essence of the review. Pacal et al.^27^ leverages powerful ViT and CNN-based deep learning models with data augmentation and ensemble techniques to achieve record-breaking cervical cancer classification accuracy on a massive dataset. This breakthrough paves the way for early and precise diagnosis, potentially reducing mortality rates and revolutionizing clinical implementation. Pramanik et al.^28^ proposes an innovative approach to enhance cervical cancer detection in Pap smear images. It introduces a fuzzy distance-based ensemble method, incorporating transfer learning models like Inception V3, MobileNet V2, and Inception ResNet V2. Additional layers are added for specific feature learning, and a unique ensemble technique is used to minimize errors. The method employs three distance measures and “defuzzification” for final predictions. By combining fuzzy logic and transfer learning, the approach aims to improve accuracy and efficiency in cervical cancer detection, potentially advancing screening outcomes. While further research is needed, initial results are promising.

Ensemble Learning, a powerful integration of baseline models, has demonstrated significant promise in reducing overfitting and improving classification accuracy. It has exhibited superiority over single models in various disciplines^29^. Nevertheless, the potential for further advancements in deep learning models for precise cervical cancer image classification remains substantial and warrants continued research and development. Table 1 summarizes the literature review based on the proposed method, utilized dataset, and attained results.

**Table 1:** Summary of Cervical Cancer Detection Literature.

Reference	Method	Dataset	Results	Remarks
Mango et al.^11^	Pap smear test + ANN model	N/A	N/A	Detection of cancerous cells in cervix.
Sukumar and Gnanamurthy^12^	MRI scans + SVM + NN	Herlev data	99.1% acc. in 2-class	Automated diagnosis using MRI scans.
Bora et al.^13^	CNN-based classification	Private dataset	Improved accuracy with feature selection	Deep CNN for image identification.
Hyeon et al.^14^	CNNs + VGG16 for feature extraction	7134 MRIs	SVM’s F1 score superior	Classifying cervical MRIs as normal or infectious.
Promworn et al.^30^	Comparative analysis of models	N/A	DenseNet161 achieved 94.38% acc.	DenseNet161 excelled among five models.
ColpoNet^15^	Inspired by DenseNet	Nat. Cancer Institute dataset	Accuracy of 81.353%	Based on computationally efficient DenseNet.
Parikshit Sanyal et al.^16^	CNN for detecting ’abnormal’ foci	1838 microphotographs	95.46% diagnosis acc.	High accuracy in classifying normal and abnormal foci.
Karunakaran et al.^17^	Ultrasensitive SERS for sample prediction	Cervix cell samples	Average acc. of 95.46%	Predicting normal, HSIL, and CSCC.
Taha et al.^18^	Pre-trained CNN architecture	Herlev dataset	99.19% acc. in 2-class	Effectiveness of pre-trained CNN architecture.
Kudva et al.^19^	Hybrid transfer learning system	AlexNet and VGG-16 features	Classification acc. of 91.46%	Improved classification with focused filters.
Xue et al.^20^	Ensemble Transfer Learning (ETL)	Herlev dataset	Highest acc. of 98.61%	ETL after developing multiple deep learning models.
Chen et al.^21^	Fine-tuned CNN architectures	4993 histology images	Achieved 97.42% classification acc.	Effectiveness of transfer learning for histopathology images.
Ghoneim et al.^22^	CNN-based approaches with ELM classifiers	Herlev database	99.5% detection acc. and 91.2% classification acc.	Utilized ELM classifiers for deep-learned characteristics in cell images.
Kang et al.^25^	Raman spectroscopy, H-CNN	Tissue samples	Over 94% accuracy in classifying tissues	H-CNN promising for precise cervical cancer diagnosis
Youneszade et al.^26^	-	Review of techniques, architectures, and segmentation methods	Overview of DL in cervical cancer screening	Emphasizes the need for further research
Pacal et al.^27^	ViT, CNN-based models,	Massive dataset	Record-breaking classification accuracy	Potential for early diagnosis and reduced mortality rates
Pramanik et al.^28^	Fuzzy distance-based ensemble	Pap smear images	Promising initial results in accuracy and efficiency improvement	Further research needed for full assessment

## Proposed method

**Fig. 1:**
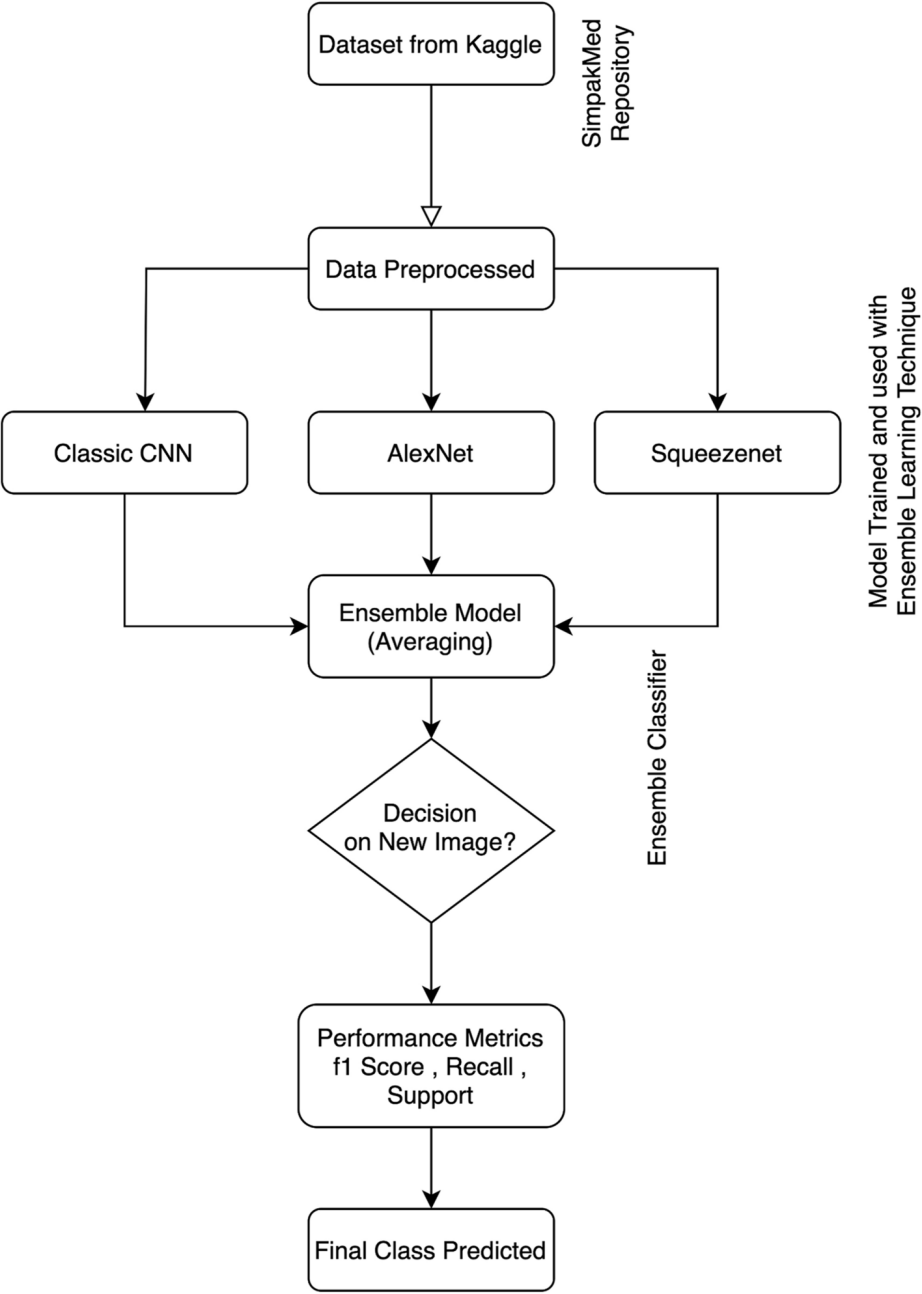
Proposed Architecture.

This section outlines the proposed methodology for this study, which primarily centers around the creation of an ensemble framework for the detection of cervical cancer. The proposed approach encompasses multiple sub processes, including image normalization, feature extraction, and model development. As base learners, CNN, AlexNet, and SqueezeNet are employed. The ensemble learning technique consolidates predictions derived from these base learners. Figure 1 displays the proposed methodology’s architecture.

### Preprocessing

In the initial phase, the input image brightness is adjusted to a range between zero and one. Through the normalization of brightness and the resizing of images, the dataset is primed for further analyses, such as feature extraction or classification algorithms. These preprocessing steps significantly contribute to the overall reliability and accuracy of subsequent stages in the study, ultimately enabling meaningful insights and conclusions to be drawn from the proposed Cervical Cancer Dataset. We resized all the images into the 64x64.

### Feature extraction

Feature extraction is a critical phase in neural network applications, playing a pivotal role in enhancing the network’s ability to discern salient patterns and information from raw data. In essence, it entails the process of transforming the input data into a more compact and informative representation, while retaining the most pertinent attributes for the task at hand. This is particularly significant in image processing tasks, as it enables the network to focus on relevant characteristics, such as edges, textures, and shapes, while discarding redundant or less discriminative information.

In the context of the proposed study, the pre-processed dataset undergoes a crucial feature extraction stage. Here, established deep learning models including traditional CNN, AlexNet, and SqueezeNet are employed. These models have garnered acclaim for their efficacy in tasks related to image categorization. By leveraging their hierarchical architectures, these models can automatically learn and extract intricate features from the input images, empowering the subsequent stages of the study with a more refined and meaningful representation of the data. This, in turn, bolsters the overall performance and accuracy of the neural network in addressing the specific challenges posed by cervical cancer detection.

### Base learner

This section discribe the detail architecture of base learer models.

#### Convolutional neural network

CNNs hold paramount importance in image classification tasks owing to their specialized architecture tailored for extracting intricate hierarchical features from visual data. This distinct architecture is composed of multiple layers that perform diverse operations on the input data^29^. In the context of this study, the input data possesses dimensions of (64, 64, 3), signifying 64 pixels in both height and width, with 3 color channels corresponding to Red, Green, and Blue (RGB).

Initiating with a 16-filter convolutional layer utilizing a (3,3) kernel and employing the Rectified Linear Unit (ReLU) activation function, the model commences its feature extraction process. Subsequently, two additional convolutional layers are introduced, each with an escalating number of filters (32 and 64, respectively) and utilizing the same kernel size as the initial layer. Following each convolutional layer, MaxPooling2D layers are incorporated to downsample the extracted feature maps, enhancing computational efficiency and reducing redundancy. To combat overfitting, a Dropout layer with a 25% rate is introduced. The output of this Dropout layer undergoes flattening, followed by processing through a fully connected Dense layer comprising 64 units and employing the ReLU activation function. Subsequent to this layer, an additional Dropout layer is applied to further mitigate overfitting risks. The final layer of the CNN encompasses 5 units, employing sigmoid activation functions to yield a probability distribution across the 5 potential classes. For optimization, the Adam optimizer is employed in conjunction with categorical cross-entropy loss, while accuracy serves as the evaluation metric. This comprehensive architecture and methodology are tailored to effectively tackle the nuances of image classification tasks, demonstrating the potency and versatility of CNNs in this domain. The detailed architecture of the CNN model is outlined in Table 4.

**Table 2:** CNN Model Architecture.

**Operation**	**Data Dimensions**	**Weights**	**Details**
Input	64x64x3		Input Layer
Conv2D	64x64x3 $\rightarrow$ 62x62x16	448	Convolution
Conv2D	62x62x16 $\rightarrow$ 62x62x16	448	Convolution
Conv2D	62x62x16 $\rightarrow$ 60x60x32	4640	Convolution
Max Pooling	60x60x32 $\rightarrow$ 30x30x32	0	Max Pooling
Conv2D	30x30x32 $\rightarrow$ 28x28x64	18496	Convolution
Max Pooling	28x28x64 $\rightarrow$ 14x14x64	0	Max Pooling
Conv2D	14x14x646 $\rightarrow$ 12x12x128	73856	Convolution
Max Pooling	12x12x128 $\rightarrow$ 6x6x128	0	Max Pooling
Dropout	6x6x128 $\rightarrow$ 6x6x128	0	Dropout
Flatten	6x6x128 $\rightarrow$ 4608	0	Flatten
Dense	4608 $\rightarrow$ 64	294976	Dense
Dropout	64 $\rightarrow$ 64	0	Dropout
Dense	64 $\rightarrow$ 5	325	Dense

#### AlexNet

AlexNet, a seminal architecture in the realm of deep learning, has been pivotal in revolutionizing image classification tasks. Comprising eight layers, it features a sophisticated arrangement of five convolutional layers interposed with three fully connected layers. The model’s input data is structured in (64, 64, 3) dimensions, indicating 64 pixels in height and width, with 3 color channels (RGB). Initiating with the first convolutional layer housing 96 filters of 11x11 dimensions and a stride of 4, the activation function employed is Rectified Linear Unit (ReLU), while padding is set to ’valid’. Subsequently, a max pooling layer with a pool size of 2x2 and a stride of 2 is incorporated. The second convolutional layer encompasses 256 filters with a kernel size of 3x3 and padding set to ’same’, followed by another max pooling layer with the same specifications as the preceding layer.Proceeding to the third through fifth convolutional layers, 384 filters with a kernel size of 3x3 and padding set to ’same’ are employed. All these layers make use of ReLU activation functions. Transitioning to the sixth through eighth layers, the architecture transitions to fully connected layers. The sixth layer boasts 4096 neurons, each employing ReLU activation functions, followed by a dropout layer with a rate of 0.5 for regularization. The seventh layer mirrors the structure of the sixth. In the eighth layer, the number of output neurons is reduced to 5, employing softmax activation functions to facilitate multi-class classification.The model is constructed employing the Adam optimizer with a learning rate of 0.0001. Categorical cross-entropy serves as the loss function, while accuracy is adopted as the evaluation metric. The detailed architecture of the AlexNet model is outlined in Table 3.

**Table 3:** AlexNet Model Architecture.

**Operation**	**Data Dimensions**	**Weights (N)**	**Details**
Input	64x64x3		Input Layer
Conv2D	64x64x3 $\rightarrow$ 14x14x96	34944	Convolution
Conv2D	14x14x96 $\rightarrow$ 14x14x96	34944	Convolution
Max Pooling	14x14x96 $\rightarrow$ 7x7x96	0	Max Pooling
Conv2D	7x7x96 $\rightarrow$ 7x7x256	221440	Convolution
Max Pooling	7x7x256 $\rightarrow$ 3x3x256	0	Max Pooling
Conv2D	3x3x256 $\rightarrow$ 3x3x384	885120	Convolution
Conv2D	3x3x384 $\rightarrow$ 3x3x384	1327488	Convolution
Conv2D	3x3x384 $\rightarrow$ 3x3x256	884992	Convolution
Max Pooling	3x3x256 $\rightarrow$ 1x1x256	0	Max Pooling
Flatten	1x1x256 $\rightarrow$ 256	0	Flatten
Dense	256 $\rightarrow$ 4096	294976	Dense
Dropout	4096 $\rightarrow$ 4096	0	Dropout
Dense	4096 $\rightarrow$ 5	4096	Dense
Dropout	4096 $\rightarrow$ 4096	0	Dropout
Dense	4096 $\rightarrow$ 5	20485	Dense

#### SqueezNet

SqueezeNet is a specialized deep convolutional neural network architecture, purpose-built for efficient and low-power inference. It is designed to achieve high accuracy in image classification tasks while minimizing computational resources and model size. The architecture commences with an input tensor of shape (64, 64, 3), representing an image with a height and width of 64 pixels and three color channels (Red, Green, and Blue).

**Table 4:** SqueezeNet Model Architecture.

**Operation**	**Data Dimensions**	**Weights (N)**	**Details**
Input	64x64x3	-	Input Layer
Conv2D	64x64x3 $\rightarrow$ 32x32x96	14208	Convolution
Max Pooling	32x32x96 $\rightarrow$ 15x15x96	0	Max Pooling
Conv2D	15x15x96 $\rightarrow$ 15x15x16	1552	Convolution
Conv2D	15x15x96 $\rightarrow$ 15x15x64	6208	Convolution
concatenate	15x15x16 + 15x15x64	-	Concatenation
Conv2D	15x15x80 $\rightarrow$ 15x15x16	1296	Convolution
Conv2D	15x15x80 $\rightarrow$ 15x15x64	46144	Convolution
concatenate	15x15x16 + 15x15x64	-	Concatenation
Max Pooling	15x15x80 $\rightarrow$ 7x7x80	0	Max Pooling
Conv2D	7x7x80 $\rightarrow$ 7x7x32	2592	Convolution
Conv2D	7x7x80 $\rightarrow$ 7x7x128	10368	Convolution
concatenate	7x7x32 + 7x7x128	-	Concatenation
Conv2D	7x7x160 $\rightarrow$ 7x7x32	5152	Convolution
Conv2D	7x7x160 $\rightarrow$ 7x7x128	184448	Convolution
concatenate	7x7x32 + 7x7x128	-	Concatenation
Max Pooling	7x7x160 $\rightarrow$ 3x3x160	0	Max Pooling
Conv2D	3x3x160 $\rightarrow$ 3x3x48	7728	Convolution
Conv2D	3x3x160 $\rightarrow$ 3x3x192	30912	Convolution
concatenate	3x3x48 + 3x3x192	-	Concatenation
Conv2D	3x3x240 $\rightarrow$ 3x3x48	11568	Convolution
Conv2D	3x3x240 $\rightarrow$ 3x3x192	414912	Convolution
concatenate	3x3x48 + 3x3x192	-	Concatenation
Conv2D	3x3x240 $\rightarrow$ 3x3x64	15424	Convolution
Conv2D	3x3x240 $\rightarrow$ 3x3x256	61696	Convolution
concatenate	3x3x64 + 3x3x256	-	Concatenation
Conv2D	3x3x320 $\rightarrow$ 3x3x64	20544	Convolution
Conv2D	3x3x320 $\rightarrow$ 3x3x256	737536	Convolution
concatenate	3x3x64 + 3x3x256	-	Concatenation
Dropout	3x3x320 $\rightarrow$ 3x3x320	0	Dropout
Conv2D	3x3x320 $\rightarrow$ 3x3x5	1605	Convolution
Pooling	3x3x5 $\rightarrow$ 5	0	Global Avg Pooling
FC	5 $\rightarrow$ 5	30	Fully Connected

It then progresses through a sequence of layers, which include convolutional and pooling operations. Notably, SqueezeNet incorporates distinctive components known as “fire modules”. These modules consist of parallel 1x1 and 3x3 convolutions, strategically designed to balance computational cost and model expressiveness. Following the fire modules, the network integrates additional convolutional layers, a dropout layer for regularization, a 1x1 convolutional layer to further refine features, and a global average pooling layer for dimensionality reduction. The architecture culminates in a dense output layer with a softmax activation function, facilitating multi-class classification. To train the model, it is compiled using the Adam optimizer, chosen for its adaptability and efficiency in minimizing the loss function. Categorical cross-entropy is employed as the loss function, which is well-suited for multi-class classification tasks. The model’s performance is evaluated based on accuracy, providing a metric to gauge its effectiveness in classifying images accurately. SqueezeNet stands out for its ability to achieve high accuracy in image classification tasks while being mindful of computational resources, making it particularly valuable for scenarios where efficiency and low-power inference are paramount. The detailed architecture of the SqueezeNet model is outlined in Table 4.

### Proposed ensemble architecture

Ensemble learning stands as a powerful machine learning technique that amalgamates multiple individual models (base learners) to yield predictions with increased robustness and accuracy compared to any single model in isolation. This technique capitalizes on the diversity inherent in these models, which may employ distinct algorithms or be trained on different data subsets, enabling them to collectively make informed decisions as shown in algorithm 1. The significance of ensemble learning lies in its ability to alleviate the limitations of individual models. By consolidating predictions from multiple models, ensemble methods often produce more reliable and accurate outcomes, leading to enhanced generalization and superior performance on previously unseen data.

In the realm of deep learning, ensemble techniques can significantly boost model performance. Deep learning models, while potent, may encounter challenges such as overfitting or difficulty in capturing intricate patterns within the data. Ensemble methods address these issues by combining diverse deep learning models, potentially featuring distinct architectures or training strategies. This approach aids in capturing a broader spectrum of features and patterns within the data, resulting in more robust and accurate predictions. In the present study, an ensemble learning approach is employed to fuse extracted features from classical CNN, AlexNet, and SqueezeNet. Leveraging the averaging methodology, a straightforward yet highly effective technique, this ensemble method aggregates predictions from each individual model to generate a final prediction. By harnessing the collective strengths of these diverse models, the ensemble approach aims to augment overall predictive performance, yielding more dependable and accurate outcomes for the given task.

**Algorithm 1:**
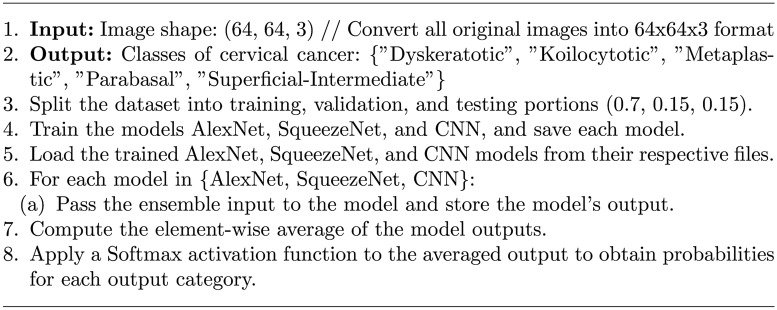
Proposed Ensemble Algorithm

## Experimental study

In this section, we present the dataset utilized in the experiment, the evaluation parameters employed to assess the model’s performance, and an analysis of the results from the proposed study.

### Dataset

The Cervical Cancer (Sipakmed) dataset stands as one of the most extensive publicly accessible resources for cervical cancer classification^31^. Comprising approximately 4096 high-resolution images extracted from pap smear tests of 715 individuals, it encompasses 70 cases afflicted with cervical cancer. These images exhibit diverse magnifications and are categorized into five groups: superficial-intermediate, parabasal, metaplastic, koilocytotic, and dyskeratotic. It serves as an invaluable asset for researchers dedicated to refining algorithms for the automated detection and categorization of cervical cancer cells. This, in turn, promises enhanced diagnostic and therapeutic approaches for cervical cancer. To facilitate effective utilization, we partitioned the dataset into three segments: training, testing, and validation, allocating 70% for training, 15% for validation, and 15% for testing purposes.

### Evaluation parameters

To assess the performance of the proposed model, we utilized various performance metrics, including accuracy, precision, recall, and F1 score. These metrics are employed to evaluate the effectiveness of the proposed methodology. In addition, graphical representations of training and validation accuracy, as well as training and validation loss, are generated. Furthermore, we conducted a comparative analysis between the proposed methodology and individual models within the ensemble, such as classic CNN, AlexNet, and SqueezeNet.

#### Accuracy

Accuracy represents the proportion of correct predictions in relation to the total test results.

1 $$\begin{aligned} \text {Accuracy} = \frac{{TP + TN}}{{TP + TN + FP + FN}} \end{aligned}$$

#### Precision

Precision signifies the ratio of correctly classified positive instances to the total instances classified as positive.

2 $$\begin{aligned} \text {Precision} = \frac{{TP}}{{FP + TP}} \end{aligned}$$

#### Recall

Recall indicates the ratio of correctly classified positive instances to the total instances actually belonging to the positive class.

3 $$\begin{aligned} \text {Recall} = \frac{{TP}}{{FN + TP}} \end{aligned}$$

#### F1 score

The F1 Score serves as a composite metric reflecting the performance of a given Machine Learning Model.

4 $$\begin{aligned} \text {F1 Score} = \frac{{2 \times (\text {Precision} \times \text {Recall})}}{{\text {Precision} + \text {Recall}}} \end{aligned}$$

Here TP denotes true positive, TN represents true negative, FN stands for false negative, and FP signifies false positive.

### Results and analysis

This section represents the results of different model. The ensemble approach proposed in this study was implemented within the PyCharm integrated development environment. This environment was configured on a system equipped with a 2.7 GHz dual-core Intel i7 processor, 16 GB of RAM, an NVIDIA GeForce ROG-STRIX graphics card with 256-bit architecture, and 8 GB of dedicated GPU memory. This robust hardware setup was chosen to ensure optimal performance during the development and execution of the ensemble model. For visualization purposes, the Seaborn and Matplotlib libraries were employed. These libraries offer a wide range of powerful tools and functions for creating informative and visually appealing plots and charts. Leveraging these visualization tools adds an extra layer of clarity and insight to the analysis, facilitating a deeper understanding of the model’s behavior and performance.

Table 5 displays the precision, recall, and F1-Score metrics for various classes, including dyskeratotic, koilocytotic, metaplastic, parabasal, and superficial intermediate, in the SqueezeNet model. Similar metrics for the CNN model are presented in Table 6, for the AlexNet model in Table 7, and for the proposed model in Table 8.

**Table 5:** Precision, Recall and F1- Score of SqueezeNet.

**Without confidence interval**				**With confidence interval (95%)**		
**class**	**Precision**	**Recall**	**F1-Score**	**Precision**	**Recall**	**F1-Score**
Dyskeratotic	0.96	0.93	0.94	0.91–0.93	0.89–0.91	0.90–0.92
Koilocytotic	0.94	0.76	0.84	0.90–0.91	0.72–0.74	0.81–0.83
Metaplastic	0.84	0.98	0.91	0.80–0.82	0.94–0.97	0.88–0.90
Parabasal	0.93	0.96	0.95	0.90–0.91	0.91–0.94	0.91–0.93
Superficial-Intermediate	0.94	0.98	0.96	0.90–0.92	0.95–0.97	0.88–0.90

**Table 6:** Precision, Recall and F1- Score of CNN.

**Without confidence Interval**				**With confidence interval (95%)**		
**class**	**Precision**	**Recall**	**F1-Score**	**Precision**	**Recall**	**F1-Score**
Dyskeratotic	0.96	0.93	0.94	0.91–0.94	0.91–0.93	0.90–0.93
Koilocytotic	0.94	0.76	0.84	0.90–0.93	0.71–0.75	0.80–0.82
Metaplastic	0.84	0.98	0.91	0.79–0.81	0.95–0.97	0.88–0.90
Parabasal	0.93	0.96	0.95	0.88–0.90	0.92–0.95	0.92–0.94
Superficial-Intermediate	0.94	0.98	0.96	0.91–0.93	0.95–0.97	0.87–0.89

**Table 7:** Precision, Recall and F1- Score of AlexNet.

**Without confidence interval**				**With confidence interval (95%)**		
**class**	**Precision**	**Recall**	**F1-Score**	**Precision**	**Recall**	**F1-Score**
Dyskeratotic	0.97	0.92	0.94	0.94–0.96	0.90–0.92	0.91–0.93
Koilocytotic	0.91	0.85	0.88	0.88–0.90	0.81–0.83	0.85–0.87
Metaplastic	0.92	0.92	0.92	0.90–0.92	0.87–0.89	0.85–0.90
Parabasal	0.92	0.97	0.95	0.86–0.90	0.90–0.95	0.85–0.88
Superficial-Intermediate	0.95	1.00	0.98	0.91–0.93	0.95–0.98	0.93–0.97

**Table 8:** Precision, Recall and F1- Score of Proposed Model.

**Without confidence interval**				**With confidence interval (95%)**		
**class**	**Precision**	**Recall**	**F1-Score**	**Precision**	**Recall**	**F1-Score**
Dyskeratotic	0.97	0.92	0.94	0.92–0.96	0.88–0.90	0.88–0.92
Koilocytotic	0.91	0.85	0.88	0.88–0.90	0.81–0.83	0.85–0.87
Metaplastic	0.92	0.92	0.92	0.90–0.92	0.87–0.90	0.86–0.90
Parabasal	0.92	0.97	0.95	0.85–0.90	0.90–0.95	0.85–0.88
Superficial-Intermediate	0.95	1.00	0.98	0.91–0.93	0.95–0.98	0.94–0.97

Training and validation accuracy plots are essential visualizations in deep learning analysis. It track the model’s performance during training and provide insights into its ability to generalize to new, unseen data. The training accuracy plot displays how accurately the model predicts the training data over epochs. Initially, accuracy may be low, but it should gradually improve. The validation accuracy plot evaluates the model’s performance on a separate validation set, which it has never seen before. This plot helps detect overfitting, as it shows whether the model is learning to generalize or simply memorizing the training data. Ideally, both training and validation accuracy should increase together. If training accuracy continues to rise while validation accuracy plateaus or decreases, it indicates overfitting. These plots are crucial for fine-tuning models, selecting the best-performing architecture, and ensuring the model’s effectiveness in making accurate predictions on new, real-world data.

**Fig. 2:**
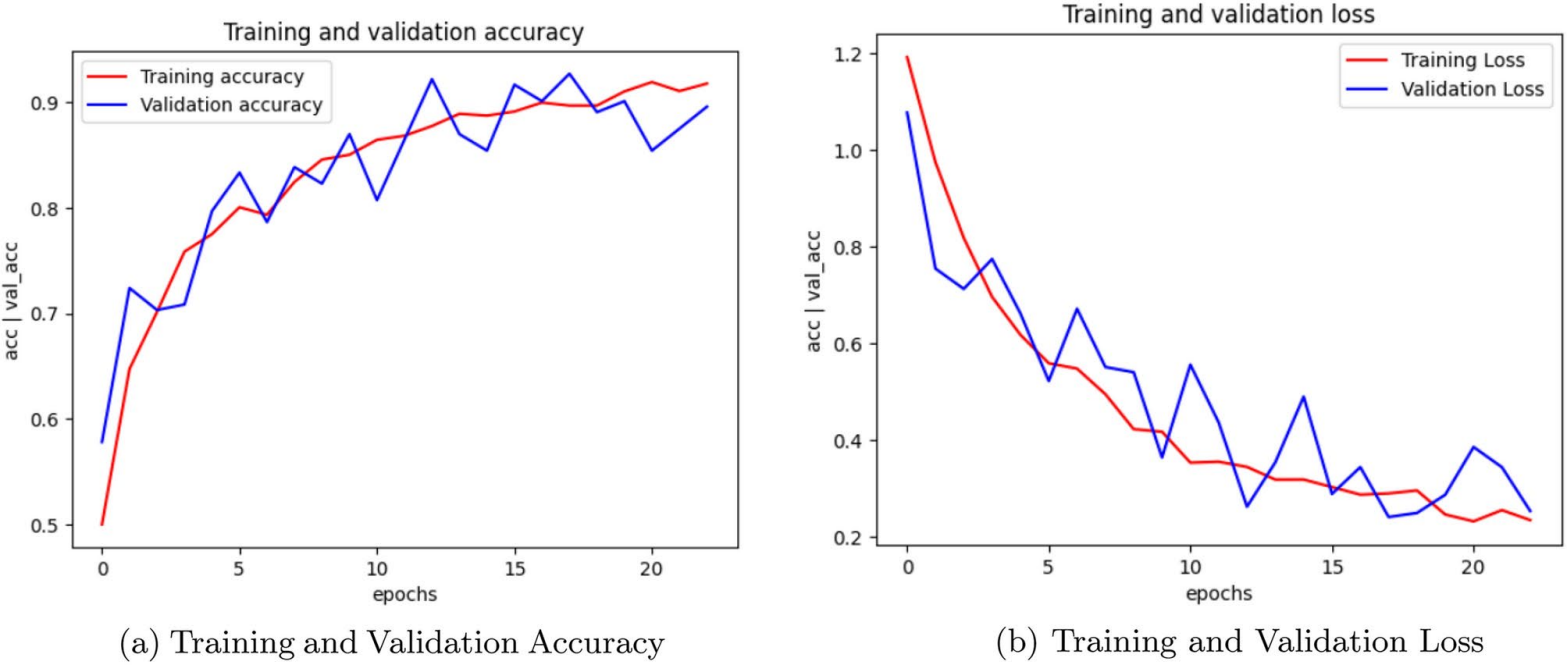
CNN Training, Validation accuracy and Validation loss.

**Fig. 3:**
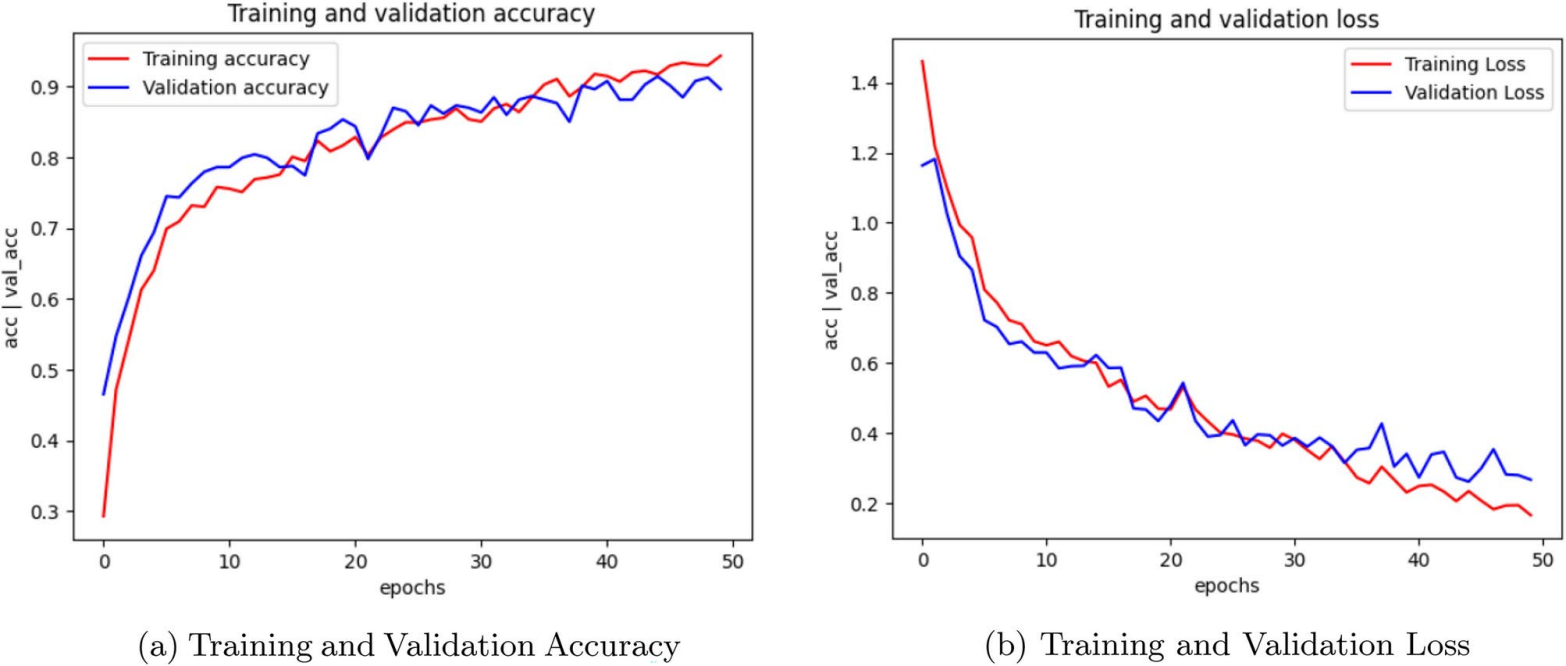
AlexNet Training, Validation accuracy and Validation loss.

Training and validation loss are fundamental metrics in evaluating the performance of a machine learning model, particularly in deep learning. The training loss measures the error between the predicted and actual values on the training data. It reflects how well the model is learning the patterns within the training set. Initially, the training loss is typically high as the model makes random predictions. As training progresses, the loss decreases, indicating that the model is becoming more proficient at making accurate predictions. Validation loss, on the other hand, assesses the model’s performance on a separate validation set, which it has never seen before. This is crucial for determining how well the model generalizes to new, unseen data. If the validation loss remains low and stable, it suggests the model is likely to perform well on real-world data. Monitoring both training and validation loss is crucial in preventing overfitting. If the training loss continues to decrease while the validation loss plateaus or increases, it indicates that the model is overfitting to the training data. Overall, tracking training and validation loss provides valuable insights into the model’s learning progress and its ability to make accurate predictions on new data. These metrics play a vital role in fine-tuning models and ensuring their effectiveness in real-world applications.

**Fig. 4:**
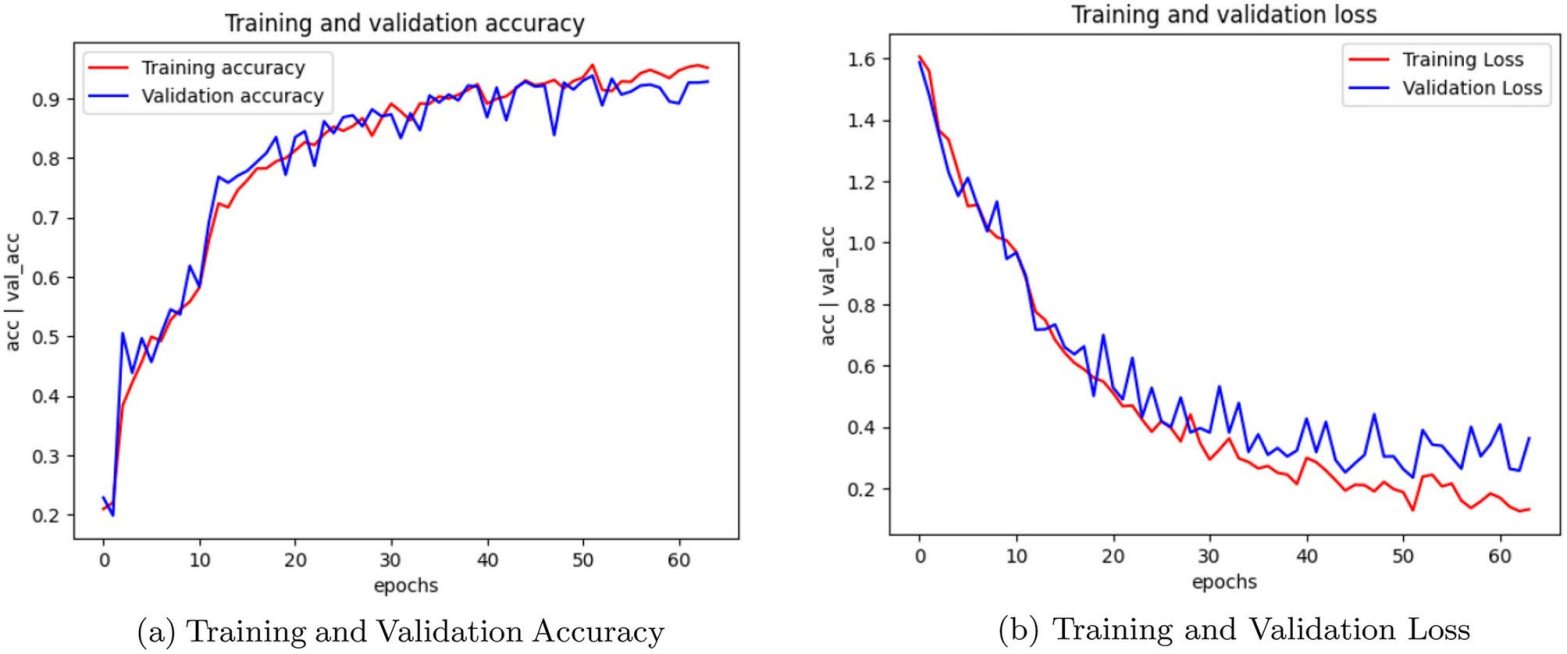
SqueezeNet Training, Validation accuracy and Validation loss.

**Fig. 5:**
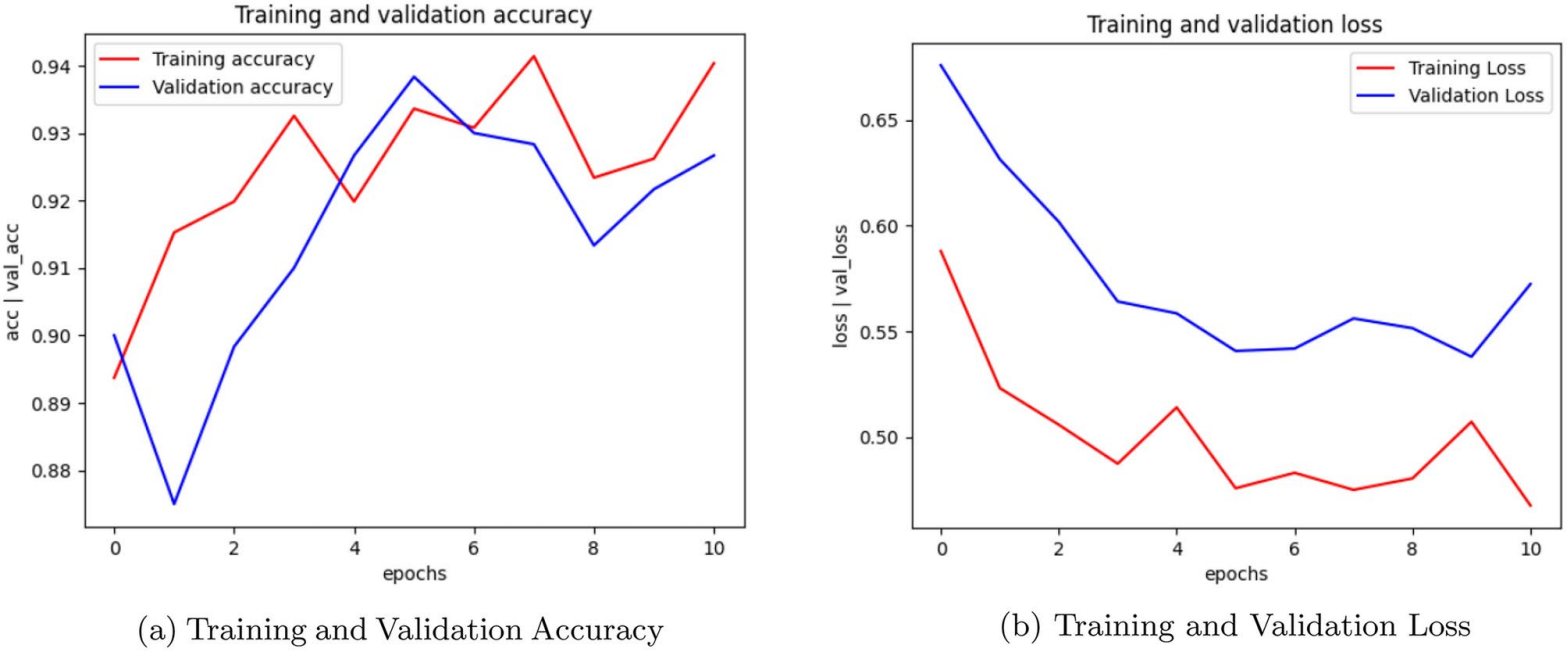
Ensemble Training, Validation accuracy and Validation loss.

Figure 2 illustrates the training accuracy, validation accuracy, and validation loss plots of CNN model. Figure 3 showcases the performance of the AlexNet model, while Figure 4 presents the results for SqueezeNet. Figure 5 provides a visual representation of the ensemble model’s performance.

**Fig. 6:**
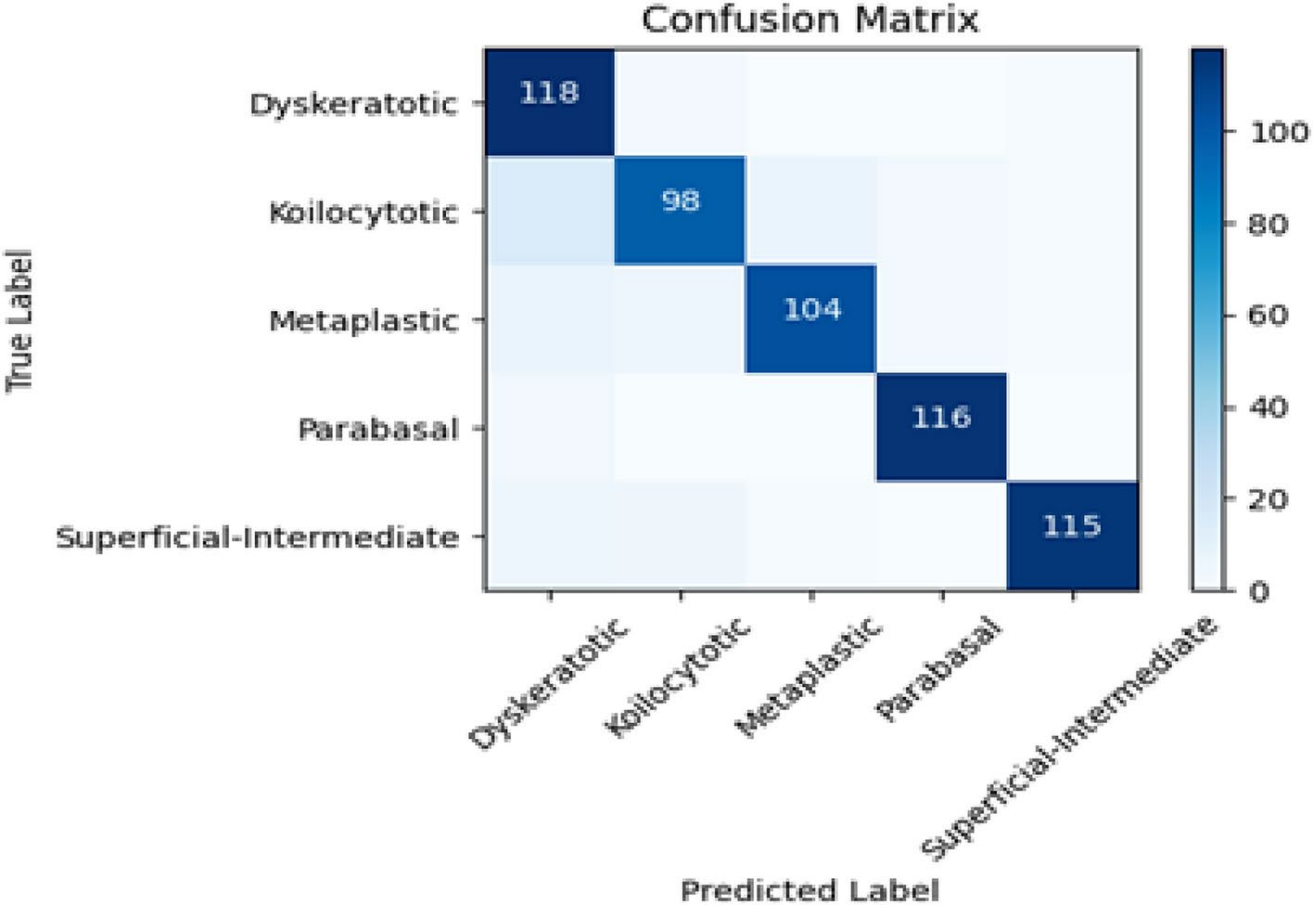
Confusion matrix of Proposed.

The presented classification report provides a concise overview of the model’s performance on a dataset comprising 586 samples distributed across five distinct classes: dyskeratotic, koilocytotic, metaplastic, parabasal, and superficial-intermediate. Accompanying this report is the ensemble model’s confusion matrix, depicted in Figure 6. Impressively, the model achieved an overall accuracy of 94%, underscoring its proficiency in accurately categorizing samples across the diverse classes

**Fig. 7:**
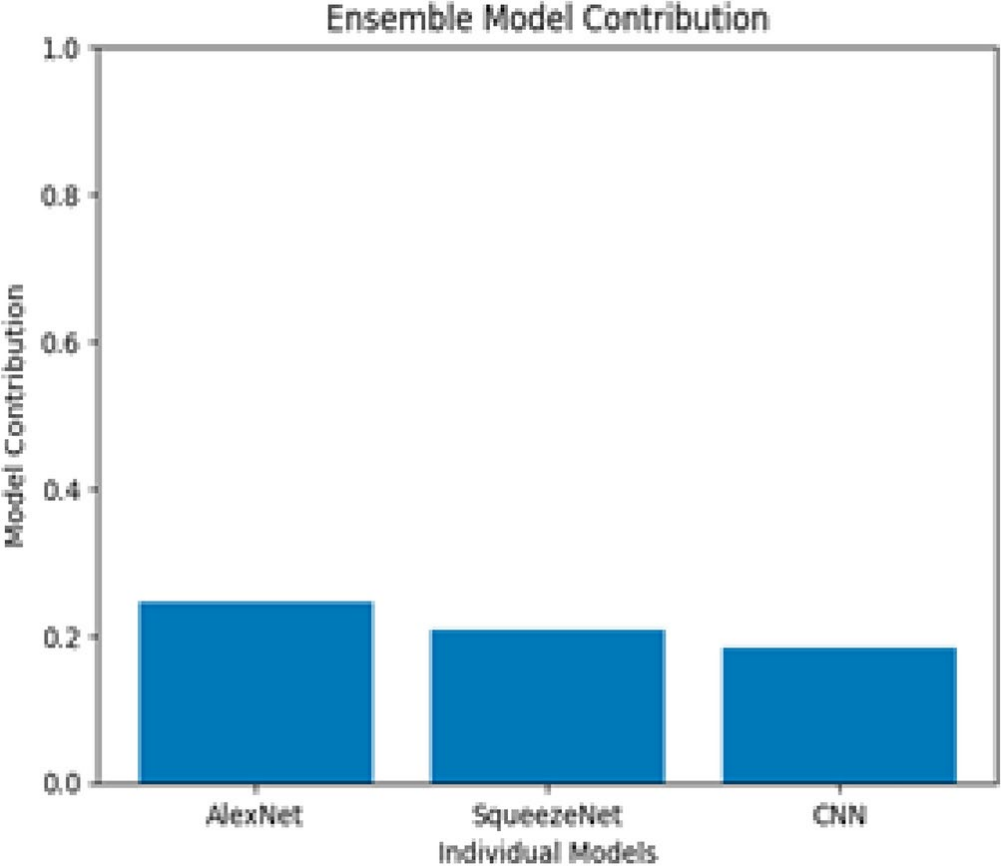
Individual Model Comparison.

Figure 7 illustrates the distinctive contributions of each base learner in the ensemble.

**Fig. 8:**
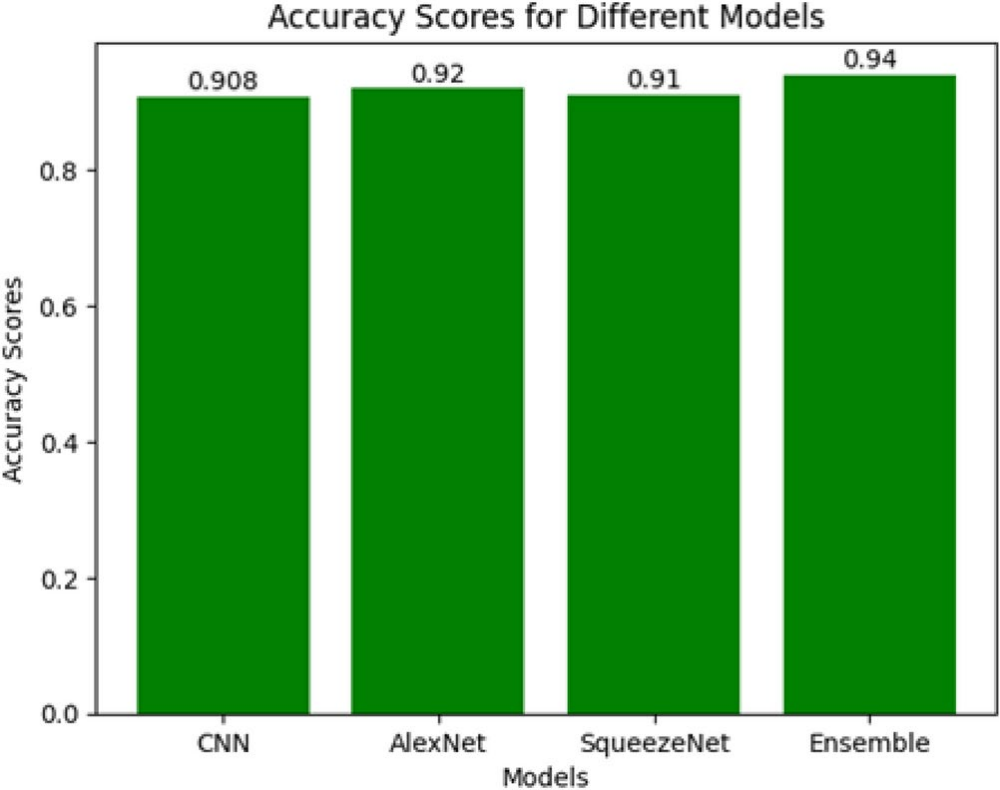
Accuracy Comparison.

Table 9 illustrates a comparison between the proposed model and other models, focusing on precision, recall, F1 score, and accuracy. The table indicates that the proposed model outperforms other models, particularly in terms of accuracy.

**Table 9:** Comparision of Proposed Model with other Models.

**Without confidence interval**					**With confidence interval (95%)**			
**Model**	**Precision**	**Recall**	**F1-Score**	**Accuracy**	**Precision**	**Recall**	**F1-Score**	**Accuracy**
CNN	0.90	0.91	0.90	0.90	0.85–0.88	0.86–0.89	0.86–0.88	0.86–0.89
AlexNet	0.93	0.93	0.93	0.92	0.89–0.91	0.90–0.92	0.88–0.91	-.87-0.90
SqueezeNet	0.91	0.90	0.92	0.91	0.88–0.90	0.85–0.88	0.87–0.90	0.85–0.89
Proposed Model	0.92	0.91	0.92	0.94	0.88–0.91	0.86–0.90	0.87–0.91	0.88–0.93

The table 10 provides a comprehensive comparison of different methods employed in the detection and diagnosis of cervical cancer, showcasing the results achieved by each method on various datasets. Promworn et al. (2019) conducted a comparative analysis of models, with DenseNet161 achieving an impressive accuracy of 94.38%. ColpoNet (Saini et al., 2020), inspired by DenseNet, achieved an accuracy of 81.353% on the National Cancer Institute dataset. Parikshit Sanyal et al. (2020) utilized a CNN for detecting ’abnormal’ foci, achieving a notable diagnosis accuracy of 95.46% on 1838 microphotographs. Karunakaran et al. (2020) employed ultrasensitive SERS for sample prediction, attaining an average accuracy of 95.46% on cervix cell samples. Kudva et al. (2020) implemented a hybrid transfer learning system, using AlexNet and VGG-16 features, resulting in a classification accuracy of 91.46%. Ghoneim et al. (2020) utilized CNN-based approaches with ELM classifiers, achieving a remarkable 99.5% detection accuracy and a 91.2% classification accuracy on the Herlev database. Kang et al. (2023) employed Raman spectroscopy and H-CNN, achieving over 94% accuracy in classifying tissue samples. The proposed method, utilizing the SipakMed dataset and Ensemble Model, demonstrated a competitive overall accuracy of 94%. While this table provides valuable insights into various approaches for cervical cancer detection, the absence of detailed information about the datasets for some methods might limit the interpretation and generalizability of the results.

**Table 10:** Results comparison with previous study.

Reference	Method	Dataset	Results
Promworn et al.^30^	Comparative analysis of models	N/A	DenseNet161 achieved 94.38% acc.
ColpoNet^15^	Inspired by DenseNet	Nat. Cancer Institute dataset	Accuracy of 81.353%
Parikshit Sanyal et al.^16^	CNN for detecting ’abnormal’ foci	1838 microphotographs	95.46% diagnosis acc.
Karunakaran et al.^17^	Ultrasensitive SERS for sample prediction	Cervix cell samples	Average acc. of 95.46%
Kudva et al.^19^	Hybrid transfer learning system	AlexNet and VGG-16 features	Classification acc. of 91.46%
Ghoneim et al.^22^	CNN-based approaches with ELM classifiers	Herlev database	99.5% detection acc. and 91.2% classification acc.
Kang et al.^25^	Raman spectroscopy, H-CNN	Tissue samples	Over 94% accuracy in classifying tissues
Proposed Method	SipakMed	Ensemble Model	Overall accuracy is 94%

The individual models perform impressively, with the CNN achieving an accuracy of 90.8%, closely followed by AlexNet at 92%, and SqueezeNet at 90%. However, when employing Ensemble Learning and combining these models through the averaging technique, an exceptional boost in overall accuracy is observed, reaching an impressive 94%, as illustrated in Figure 8. This amalgamation of models demonstrates the power of ensemble techniques in harnessing the strengths of diverse learners to achieve superior predictive performance.

## Discussion

The study presents a robust approach to cervical cancer classification using deep learning techniques.The integration of multiple CNN architectures through ensemble learning significantly improves classification accuracy, demonstrating the potential for more reliable and accurate diagnosis.The individual models (CNN, AlexNet, and SqueezeNet) and the ensemble model achieved high accuracy rates, suggesting the effectiveness of the proposed approach.The study’s focus on classification of squamous cells into distinct classes provides a valuable tool for clinicians to assess disease severity and guide treatment decisions.The dataset used in the study, while comprehensive, may not fully capture the diversity of real-world cervical cancer cases. A larger and more diverse dataset would be beneficial for training more robust models.The study primarily focuses on the technical aspects of the model. Further validation in a clinical setting is needed to assess the practical impact of the model on patient outcomes.While deep learning is a powerful tool, it can be computationally expensive and requires specialized hardware. Exploring simpler, more efficient models may be necessary for resource-constrained settings.By addressing these limitations and building upon the strengths of this study, future research can further advance the field of computer-aided diagnosis for cervical cancer.

## Conclusion

Incorporating Classic CNN, AlexNet, and SqueezeNet models through ensemble learning demonstrates robust efficacy in detecting squamous cells and assessing cervical cancer severity. Individually, AlexNet achieves the highest accuracy at 92%, with all models performing commendably. However, ensemble integration further boosts accuracy to an impressive 94%. This approach addresses the need for more efficient cervical cancer detection, especially in less developed regions. Categorizing squamous cells into distinct groups greatly aids in assessing cancer gravity for targeted treatment, promising improved patient outcomes. While significant, there’s room for refinement. Future work may focus on enhancing model accuracy and exploring additional deep learning algorithms. Limited public datasets presently constrain accuracy, but amassing a dedicated cervix cancer dataset and developing a new deep learning model holds promise for substantial progress in medical image processing and enhanced cervical cancer detection and treatment.

## Data Availability

Dataset used in the experiments can be found on: https://www.kaggle.com/datasets/prahladmehandiratta/cervical-cancer-largest-dataset-sipakmeds
